# It’s not all about price: factors associated with roll-your-own tobacco use among young people - a qualitative study

**DOI:** 10.1186/s12889-018-5921-8

**Published:** 2018-08-08

**Authors:** Elizabeth Breslin, Joan Hanafin, Luke Clancy

**Affiliations:** TobaccoFree Research Institute Ireland (TFRI), Focas Research Institute, DIT Kevin Street, Camden Row, Dublin 2, Ireland

**Keywords:** Smoking, Roll-your-own cigarettes, RYO, Tobacco, Incentives, Dis-incentives, Young people, Qualitative research, Ireland

## Abstract

**Background:**

Smoking prevalence in Ireland is falling in all age groups, but the prevalence of roll-your-own (RYO) tobacco use is rising among young people. This qualitative study aims to explore and understand the factors associated with young people’s use of RYO products.

**Methods:**

Semi-structured individual and focus group interviews were conducted with young people aged 16–22 years. Participants were recruited from a higher education institution and youth organisations working with early school leavers across Dublin. In total, there were 62 participants in the study, consisting of 22 individual interviews and eight focus group interviews with 40 participants. Categoric and thematic data analysis was used to generate the findings.

**Results:**

We identified two broad themes, incentivising and disincentivising factors. The lower cost of RYO products compared to pre-manufactured cigarettes was the most important incentive for users. However, other product characteristics, such as the artisanal factors associated with RYO products were also found. Social and environmental influences were apparent, in which certain groups and environments facilitated and normalised RYO practices. Amenities and facilities often provided smokers with normalised spaces which could be dedicated to the enactment of rolling practices and to the creation and maintenance of social bonds with other users. Disincentives included negative features related to the product itself, adverse health effects, and the effects of tobacco denormalisation.

**Conclusions:**

While the lower cost of RYO products is very important for young smokers, other product characteristics and influences also incentivise and disincentivise use. A more comprehensive understanding of the multi-dimensional appeal of these products will assist policymakers to target strategies to reduce the attractiveness to young smokers of these products.

**Electronic supplementary material:**

The online version of this article (10.1186/s12889-018-5921-8) contains supplementary material, which is available to authorized users.

## Background

Ireland has a wide range of legislative measures and health promotion policies [1] to reduce the numbers of people smoking (Additional file 1). Prevalence rates of smoking in the general population have been falling steadily from 31% in 1998 [2] to 17.6% in 2017 [3]. Despite this decrease, levels of roll-your-own (RYO) tobacco use, which have been found to be at least as harmful [4] as pre-manufactured cigarettes, are on the rise among smokers in Ireland [5]. RYO cigarettes are made using loose tobacco and cigarette papers and can be smoked with or without a filter. In the general population, these products have seen a rise in use from 2.3% in 2002 to 29.5% in 2017, and among smokers under 25 years from 1.8% in 2002 to 50.6% in 2016. Recent findings show that, for the first time among adolescent and young adult smokers in Ireland, the use of RYO tobacco (11.8%) has overtaken the use of pre-manufactured cigarettes (10.9%) [6]. High levels of RYO use have also been found among young smokers in the United Kingdom [7] and New Zealand [8].

Cross-sectional population surveys examining the rise of RYO use among smokers are limited but growing. The lower cost, taste preferences, perceived lower health risks, greater satisfaction compared to pre-manufactured cigarettes, and the belief that RYO products allow users to reduce the amount smoked have all been identified as motivators for use among adult smokers [8, 9]. Demographic characteristics have also been identified, with studies highlighting that RYO users are most likely to be young, male and from lower socio-economic groups [5–7, 10].

Qualitative studies exploring RYO practices among adolescent and young adult users are rare. However, a recent New Zealand study [12] focusing on 18–30 year olds found similar reasons for RYO use as those outlined above for adults aged 18 and over [9]. While previous studies have reported on the growth of RYO use and the array of favourable product characteristics for users of these products, there has been minimal research carried out which explores users’ experiences beyond these product features. No research, to our knowledge, has considered negative aspects associated with RYO products. A comprehensive understanding of how these products incentivise and disincentivise use is important so that strategies can be developed to reduce their use. In this study, we report our findings from a qualitative study of RYO use among adolescent and young adult smokers in Dublin, Ireland. This study was undertaken following a survey which found a high prevalence of RYO use among 16–17 years old students in Ireland [6]. A qualitative study was used as a follow-up in order to understand young people’s RYO use in more depth. In particular, it was hoped that a qualitative study would provide richer data in relation to price as an incentivising factor and additionally to generate other data about why young people use RYO, and what they like and dislike about RYO. While some attitudinal factors were captured in the survey, the qualitative individual and focus group interviews generated more in-depth and organic data that were not possible to capture within the limits of a quantitative survey.

## Methods

### Approach and rationale

A qualitative approach was adopted in this study in order to explore the reasons behind the growing levels of RYO use in Ireland. The idea for this study was conceived by the third author (LC) and KB after their previous survey research found high levels of RYO use among Irish adolescents [6]. The survey, which used a cross-sectional design, to survey adolescents found increasing levels of RYO use among adolescent Irish smokers. While growing levels of RYO levels were found among this group, uncertainty remained as to the reasons behind RYO use in adolescent users. As qualitative research prioritises process rather than outcome and allows for an emphasis on meaning [13], it was deemed an appropriate approach for this study as it allowed for the generation of understanding about a specific life experience, namely how young people deal with, and make sense of, being a RYO smoker.

### Sample and recruitment

Participants in the study were aged between 16 and 22 years and comprised two groups. One group (three centres- 34 students) was recruited through a youth organisation which works with early-school-leavers from disadvantaged areas. The other group (two centres- 28 students) comprised students attending a higher education institution in Dublin City Centre.

The age range (16–22 years) was chosen as this study was a follow-on from a previous Irish survey [6] which found a high prevalence of RYO use among students 16–17 years old. This study sought a more in-depth account of RYO use from a young age group that accesses tobacco products. None of the higher education students was under 18 years and only a small number of the early school leavers was and we did not wish to exclude these, particularly as the early school leaving group is an important group to reach in terms of RYO use, as almost all are from lower socio-economic groups. Gender and age data were available to the researchers although the proliferation of themes and sub-themes from the inductive analysis (below) was such that they did not emerge as notable distinguishing features.

All of the participants had smoked a RYO cigarette within the previous 30 days. Eight focus group (40 participants) and 22 individual interviews were conducted. The individual and focus group interviews with the higher education students were conducted in classrooms within these institutions (two sites). The interviews and focus groups with the early school leavers took place in youth organisations across Dublin (three sites). Table 1 provides an overview of the two groups and the data collection methods associated with each group.

**Table 1:** Sample and data collection methods

The Sample			
Group 1	Early School Leavers (ESL)	This group of participants was recruited through a youth organisation working with early school leavers in disadvantaged areas of Dublin	
Group 2	Higher Education Students (HES)	This group of participants was recruited through a higher education institution in Dublin City Centre	
Data Collection Methods			
Interviews (INT)	Number of Participants		Participant (Pseudonym)
HES	10	6 Male (M); 4 Female (F)	Finn (M); Áine (F); Daisy (F); Davis (F); Quinn (F)
ESL	12	6 Male; 6 Female	Catriona (F); Liffey (F); Marnie (F)
(Sub-Total)	(22)		
Focus Groups (FG)			
FG-A	7	HES (M)	Joe; Marcus; Tadgh; Paul
FG-B	6	HES (F)	Brenda; Lisa; Sally
FG-C	6	ESL (M)	Andrew; Robert
FG-D	6	ESL (F)	Deirdre; Katie; Natalia
FG-E	3	HES (M)	Derek
FG-F	2	HES (F)	Anna; Kate
FG-G	5	ESL (M)	Daniel; Kyle
FG-H	5	ESL (F)	None
(Sub-Total)	(40)		
Total	62		

### Data collection

Individual and focus group interviews were used to elicit participants’ views using a prepared guide which contained questions focusing on participants’ initiation experiences, feelings towards RYO products, brand preferences and user patterns. (Participants were also questioned about smoking in general and about e-cigarette use but only the data about RYO use are presented in this paper.) The questions contained within these guides focused on learning about the behaviours and opinions of young people towards RYO products. In particular, the questions centred on understanding the experiences of ‘why’, and ‘how’ the young people used these products; ‘what’ they liked or disliked about the products, ‘who’ did they use them with, and ‘where’ did they use them. The individual and focus group interview guides were created to give flexibility to the participants so that their responses could be probed and explored fully. On average, interviews lasted 20 min, with focus group interviews running up to 60 min. A copy of the individual and focus group interview guides can be found in (Additional file 2).

### Ethical issues

Ethical approval was received from the Dublin Institute of Technology’s (DIT) Research Ethics Committee. Informed consent was given by all participants and participants were assured of confidentiality. In keeping with that, only pseudonyms were used. No participant’s real name was used at any stage as real names were replaced by pseudonyms in the transcripts and only these pseudonyms were used during data analysis and drafting of the paper.

We used pseudonyms to allow the reader to track and differentiate the quotes across different participant responses. Additionally, the use of pseudonyms “humanises” participants which we believe is important in qualitative research, while offering confidentiality and not compromising participants’ identity. In reporting participants’ (pseudonymous) quotes, we also use participants’ gender and educational affiliations.

### Data analysis

A thematic analysis approach was employed to analyse the data [14]. The recordings from the individual and focus group interviews were transcribed verbatim. The analysis of each data set began with each transcript being printed and entered into a ring-binder folder. The transcripts were uploaded to NVivo 11 by the first author and preliminary codes generated. All transcripts were read and annotated (in print and digitally) by each of the three authors. A meeting of all three authors was held to discuss points of interest from the coding and the annotation of the transcripts. Using an inductive analytic approach, the second author gathered together the low-level, descriptive codes that resulted from the NVivo coding and the annotations from the transcripts to form conceptual categories (Categoric analysis). From there, the second author generated a number of interpretive themes and sub-themes (Theoretical analysis). These themes and sub-themes framed the overall analytic structure and shaped the presentation of findings. Regular meetings were held with the three authors to discuss and refine the themes; and in particular, the structuring of the sub-themes for the final analysis and drafting of the manuscript.

## Results

Two main themes emerged from the data, categorised as incentivising and disincentivising factors associated with RYO tobacco use. The sub-themes within incentivising factors included intrinsic features of RYO; peer and familial influences; and environmental factors. Disincentivising factors related to product; health; and denormalisation. Table 2 provides an overview of the themes and sub-themes arising from the study.

**Table 2:** Overview of findings

Incentivising Factors Associated with RYO use	
I. Cost	
II. Intrinsic features of RYO	
a. Ritual and artisanship	
b. Product characteristics	
c. Brand and packaging characteristics	
d. Perceived healthier alternative	
III. Social Factors	
a. Peer influences	
b. Parental and familial influences	
IV. Environmental factors	
a. Home environment	
b. Education centres	
c. Leisure venues (pubs, nightclubs)	
Disincentivising Factors Associated with RYO Use	
I. Recent price increases	
II. Negative product characteristics	
III. Health effects	
IV. Denormalisation	

### Cost of RYO products

The lower cost of RYO products compared with pre-manufactured cigarettes was the most strongly reported motivation for RYO use. Smoking initiation for nearly all of the participants was with pre-manufactured cigarettes. Over time, the cost of pre-manufactured cigarettes was seen as too high, leading to RYO products becoming the favoured alternative:

You’re not spending €10 on a box, that’s just ridiculous (Joe, Male, HES).

### Intrinsic features

Apart from lower cost, the main incentivising factors for RYO use related to characteristics intrinsic to the RYO tobacco product itself.

#### Ritual and artisanship

Rituals and artisanal properties were associated with the RYO experience. Constructing the cigarettes was viewed as a learnt “skill” which required guidance and practice but one which was looked upon favourably:

…the thing that I like about [it is] I actually have to go and make it and I think that is a major attraction for me to roll (Finn, Male, HES).

The higher education students, in particular, discussed how they enjoyed making the cigarettes for the “meditational” and “therapeutic” benefits:

It’s also therapeutic to sit down and roll because, whereas with cigarettes you can just quickly put one in your mouth, there is kind of a ritual associated with rolling tobacco. You have to take it [tobacco] out, you have to start rolling it and so you go through the motions (Derek, Male, HES).

Once mastered, the ability to roll cigarettes themselves provided users with a sense of accomplishment and pride. Some participants elevated the satisfaction gained, labelling it “an art-form”.

While pre-manufactured cigarettes offer a more convenient smoking experience, which was in many cases favoured by the early school leavers, pre-manufactured cigarettes fail to offer the perceived social benefits associated with RYO products which were especially important for the higher education students:

I was just rolling myself a cigarette and someone came over to me and asked if I had a spare cigarette, so he started rolling his and then we started talking, I associate smoking [RYO] with making new friends (Derek, Male, HES).

#### Positive product characteristics

Overall, participants described RYOs as stronger, having “more of a hit”, with the main brand being often described as tasting “harsh”. With the rationale of the lower cost in mind, participants were able to justify the taste and strength differences, with many developing RYO preferences over time:

They’re completely different strengths…You get used to smoking a rollie and it feels like it is much stronger… like everything else, you get used to something and you stick with it (Áine, Female, HES).

Participants discussed how they increase the strength of RYO by using “roaches” (i.e. cardboard tips) instead of filters in order to “commando” it (Robert, Male, ESL).

Descriptions of taste were quite detailed in relation to heat, mildness and sensation:

Moist tobacco: that’s why I smoke rival brand 4 [alternative brand to main brand]. I don’t like an intense taste. It’s more the sensation of the inhalation. So something mild, kind of moist. I hate dried tobacco- it hurts your throat (Finn, Male, HES).

The range of choices of taste and strength in different RYO products gave rise to strong reported brand loyalty. For example, students would point to the inferiority of rival brands and assign derogatory taste attributes to them, such as labelling them “airy” [rival brand2] and “rotten” [rival brand3].

#### Brand and packaging

Brand awareness and loyalty were strong, acting as further incentivising factors in RYO use. Brand at initiation was a good predictor of current product use. *Main* brand was favoured by the majority of participants and was discussed positively in terms of its cost, availability, popularity, and range of in-brand variation:

I wouldn’t touch anything that wasn’t [main brand]. It just depends, it can either be personal preference, or it can be money or it can be some combination between the two (Daisy, Female, HES).

As evidence of their brand loyalty, participants described going to significant lengths in order to purchase a box of their preferred tobacco:

…Last week at one point; I think I went to four shops to find a box of [main brand] (Quinn, Female, HES).

Packaging played an important role in building brand preferences and loyalty:

The box comes with the filters and the skins…I like the convenience of the box; it’s so much handier than a pouch (Quinn, Female, HES).

Packaging was identified as important for brand loyalty in rival brands [rival brands] as much as in the main brand:

[Main brand] used to always dry out and taste horrible…but the [rival brand] didn’t because you just reseal it (Kate, Female, HES).

Pack colour was invoked both positively and negatively in terms of brand loyalty:

One of my friends gets [rival brand 1] because the packaging was green and that was his favourite colour (Derek, Male, HES).

I wouldn’t be able to get [main brand] because of the yellow box (Katie, Female, ESL).

The different quantities available cater for different situations or financial budgets:

I’d buy the box [12.5 gram], [but] if I was going to a festival I would buy the pouch [50 gram] (Áine, Female, HES).

#### RYO perceived as a healthier alternative to pre-manufactured cigarettes

The majority of participants recognised the health risks associated with smoking. However, the participants perceived RYO cigarettes to be a healthier alternative to pre-manufactured cigarettes RYO products were said to offer a more “natural”, less “synthetic”, “fresher” smoking experience with participants perceiving handmade cigarettes to have “less tar” and “less chemicals” than pre-manufactured cigarettes:

…smoking is not good for you, but rollies in some ways are better… as there is a lack of certain chemicals in it. If you take apart a cigarette, it looks like sawdust; whereas when you have rollies, it’s fresh, it’s soft; it’s tobacco (Daisy, Female, HES).

The visibility of the contents of RYO products contrasted with pre-manufactured cigarettes and there was a suggestion that pre-manufactured cigarettes might contain toxins or chemicals intended to make them burn more quickly:

I think the tobacco in cigarettes [has more] chemicals, because with rollies we know what we’re putting into the smoke; there could be anything in your [pre-manufactured cigarettes] smokes. And it burns so much quicker as well, there has to be a reason for it (Brenda, Female, HES).

The participants perceived that due to the time-consuming nature of the rolling process, they would smoke less than with pre-manufactured cigarettes:

I switched to rollies because it is really time-consuming, so you do have to go and make the effort…I could probably smoke two smokes in the time I’d smoke one rollie (Brenda, Female, HES).

Another perceived health benefit of RYO cigarettes related to having the ability to “moderate” the amount of tobacco used within each cigarette:

So I would pack a rollie…so you smoke less because first of all, you have to roll and second of all, you get a stronger kind of drag off it (Daisy, Female, HES).

Finally, RYO cigarettes self-extinguish when they are not inhaled, which contrast with pre-manufactured cigarettes which continue to burn slowly:

[an] upside [to] a rollie is that it goes out…there is no waste (Anna, Female, HES).

### Social - peer and familial influences

#### Peer influences

The majority of the participants were introduced to RYO by their friends who often recommended RYO as a cost benefit:

I was in first year [in college] and I didn’t have the money…and someone was like, would you not smoke rollies? (Quinn, Female, HES)

Peers could make introductions to a particular brand type which they smoked themselves:

I got a smoke off one of the lads who rolled me one, it was actually a rollie and it was [main brand] but it was the really strong type…after that I think I got a Christmas present off someone in college, I got a [main brand] light (Lisa, Female, HES).

Peers acted as demonstrators to new users of the rolling process:

They were like "we can help you roll if you need to be able to roll", so when I came [to college], I just started going with the rollies (Quinn, Female, HES).

Over time, users were able to establish friendships through their rolling practices:

It is a community as much as it is a place to smoke. There are a lot of people there who come together just to talk about stuff that’s happening and have a cigarette (Joe, Male, HES).

Once established within this community, participants felt that it was often difficult to disengage from their smoking peers, which reinforced their smoking habits:

If I wasn’t to go out [to the smoking area], I would be left on my own because all of them smoke (Marnie, Female, ESL).

Some participants explained that, if their friends suggested quitting, they would discourage and even possibly block any attempts to stop:

I just felt awkward around her because I’m so used to seeing her smoke… it was like she was different and I wanted to tell her to go home. I ended up saying, ‘here you better take a smoke or get out’…Anyway, she ended up taking a smoke and she got back smoking (Deirdre, Female, ESL).

#### Parental and familial influences

Most participants who lived at home and who came from homes where there were smokers present, reported that smoking was normal, visible and an almost inevitable part of their lives. This led many participants to feel that they were “constantly around smoking” (Natalia, Female, ESL).

Both my parents smoke so I kind of knew from the smell kind of, what they would taste like. I kind of think it was just in me to smoke (Quinn, Female, HES).

Some explained that their parents felt that they could not object to their smoking as they were smokers themselves:

I told her [mother] after a while when I started and she was like, “oh I was younger than you”, she was like, “I can’t give out to you because you’re older” (Marnie, Female, ESL).

One participant described how she made her father’s RYO cigarettes when she was younger. This experience was described positively as she felt she was helping her father:

I remember watching my dad…and then I’d be like; can I make you a few? And he used to let me make him some, and he thought it was great so that he didn’t have to make them (Deirdre, Female, ESL).

Relationship building and bonding were important in the context of smoking behaviours. Elements identified included opportunities for spending time together, conversation, learning to roll, a commonality of interest, shared activities, and even co-purchasing. These influencing groups, combined with intrinsic product characteristics, create favourable conditions that incentivise RYO use.

### Environmental factors

Physical structures had a role in incentivising RYO use, in particular, features of users’ domestic environments, facilities in education centres, and amenities in social areans such as pubs and clubs.

#### Home environment

For some participants, smoking occurred in domestic settings. Where smoking was not permitted in the home, RYO users either did not smoke at home or availed of outdoor spaces such as the garden:

My mam really hates smoking… [so] I don’t smoke in the house ever, literally never [but] if I am in the garden, she doesn’t care (Finn, Male, HES).

Where there were other smokers present, the existing “smell of smoke” was seen as a feature of the space that made it easier to smoke:

I go outside to have a smoke…I go out with my mam and talk to her. She’s smoking so I’d just have a smoke (Liffey, Female, ESL).

In domestic settings where RYO users were not permitted to smoke indoors, they reported that sometimes they did smoke indoors but concealed it:

I smoke out my window because they smoke out the kitchen door which is below my bedroom. Whenever I leave my bedroom window open you can smell the smoke below anyway (Quinn, Female, HES).

In other cases, they identified health issues, or a desire to avoid conflict, or respect as reasons for not smoking indoors:

I would never smoke in the house. I have smoked outside the house (in the garden) but only when they are not around. (Joe, Male, FG, HES)

Yeah, like one o’clock in the morning, they’re in bed; let’s go for a smoke, finally. (Paul, Male, FG, HES)

And why wouldn’t you smoke in front of your parents? (Interviewer)

Because I don’t want to disappoint them. (Paul, Male, FG, HES)

And because there is a respect thing there (Joe, Male, FG, HES)

#### Facilities in education centres

Four of the five education sites where the research was carried out had identifiable smoking areas which were either formally or informally designated. The smoking area was formally designated in one higher education site; called the “shed” [a three-sided structure covered by a roof, and containing a built-in bench with ashtrays mounted on the walls]. The smoking spaces were informally designated at early school leaver sites, at the periphery or back of the building, generally out of sight but which they could easily access, in proximity to the students’ classrooms.

##### ‘The shed’

The shed was a particularly important environment for facilitating RYO use among the higher education. The shed was described as a “fun” area in which friendships were formed and social bonds were strengthened:

The people that are down in the little shed down there, they basically are my friends in college. I don’t really know anyone else (Quinn, Female, HES).

The shed was seen as a place to switch off and to partake in the ritualistic behaviours associated with RYO products:

Even if I was to go over [to the smoking area] and not do anything [smoking], I’d still be thinking about everything. I think it’s the act of actually rolling, chatting to someone else, and usually that person is smoking; you completely switch off (Tadhg, Male, HES).

The shed was used before, between, instead of, and after classes:

I find I smoke more in college than if I was sitting at home, because … the way there might be a break between two lectures so you could either stay in or else buzz out with your friends, having a chat, having the craic and then having a smoke (Sally, Female, HES).

#### Leisure venues (pubs, nightclubs)

Participants reported that smoking was facilitated in various ways in leisure venues such as pubs and nightclubs. Many of the participants spoke about how they and their friends often felt more compelled to smoke in social situations where there was alcohol involved:

I wouldn’t have really smoked properly until I was about 16 or 17 when I started probably going out but yeah definitely it was mostly to do with drinking that I started smoking properly you know (Áine, Female, HES)

The participants discussed being able to identify ‘good’ smoking areas, with the criteria being based on the amenities and facilities offered:

They’ve heaters and all out there for you. You’d spend more time out there than you would inside…it wouldn’t be as loud…and you can still hear the music (Daniel, Male, ESL).

These areas provide opportunities to meet new people, with many participants feeling that it was easier to have fun, to talk with friends within these areas:

…In the club, there’s loud music and you’re either dancing or you’re drinking and if you want to have any conversation, you go out to the smoking area; so you’re in the smoking area, and people are smoking so you might as well (Paul, Male, HES).

Participants reported that these areas enabled an increase in the amount smoked, especially if there was alcohol involved:

Last night my friends and I were in the [licenced premises] and between four of us, we went through two boxes of rollies and a box of [pre-manufactured cigarette brand] so if you’re drinking, if you’re outside in a smoking area, you just don’t stop (Tadhg, Male, HES).

Formal and informal smoking areas incentivise smoking through designation, location, structure, amenities and facilities. These spaces are associated with friendships, comfort, and shared practices. They have an additional ready-made community with a common focus, and they add to the important relationship building elements described above. These relational factors which are enabled by designated smoking shelters encourage increased consumption of tobacco, according to participants in this study.

Smoking in general is facilitated by environmental factors such as smoking areas in education facilities and outside areas of pubs and clubs where alcohol is served. While these areas exist to facilitate smoking, they have an extra role in RYO tobacco use by providing the shelter, space and time necessary to construct RYO cigarettes. We have heard from participants in this study that, compared with smoking pre-manufactured cigarettes, RYO use is an activity that demands that the user engage in a dedicated activity, out of the wind and rain, and preferably standing still. This more static experience involved in constructing RYO cigarettes is facilitated by the dedicated space, shelter and time that smoking areas provide. In this regard then, environmental factors identified in this section particularly incentivise RYO use.

### Disincentivising factors

There were a number of disincentivising factors associated with RYO including recent price rises, negative product characteristics, perceived health effects, and denormalisation.

#### Recent price increases

The rising cost of RYO products brought about by the Irish government policy of increasing taxation on RYO tobacco was seen as a disincentivising factor by participants. As the lower cost was the key motivating factor underlying initiation in the first place, increasing prices of RYO tobacco was viewed negatively:

They’re going up in price. When I first started smoking, they were like €4.20 a box, and now it’s like €6.50. It’s crazy (Paul, Male, HES).

#### Negative product characteristics

Certain product issues, such as the “unequal balance between filters and skins” (Sylvia) within the ‘smoker’s kit’ as well as issues with the filters were considered undesirable:

Sometimes the filter can get really soggy and it’s disgusting (Kate, Female, HES).

The ‘effort’ associated with creating the product and construction difficulties at certain times [e.g. when it’s “lashing rain” (Brenda, Female, HES)], or at inconvenient times [e.g. “when you’re out of yer head” (Andrew, Male, ESL)] also inhibited users’ experiences. However, participants explained how they often enacted strategies in order to minimise such disruption. Some participants would ask their friends to roll the cigarettes on their behalf, with others purchasing pre-manufactured cigarettes for certain occasions, while some participants would pre-roll in advance:

I’ve definitely done it before, like pre-rolled maybe three smokes just if you know you’re going to be too twisted [drunk]. I’ve been twisted so many times that I actually can’t roll (Brenda, Female, HES).

Some of the early school leavers discussed feeling embarrassed by RYO products, which was not discussed by the higher education students. Specifically, some of the early school leavers discussed how they often felt “judged” due to the unattractive nature of the rolling process:

Because it’s just…not very attractive you know? Taking, licking it and putting tobacco- like no! People are looking at you and you just feel judged (Caitriona, Female, ESL).

Other participants explained the product itself made them feel embarrassed, as they perceived it to be an inferior substitute for pre-manufactured cigarettes:

It’s not even the same tobacco, it’s like stringy, it’s like smoked tobacco [pre-manufactured cigarettes] is ground and then tobacco you buy in a pouch is pure string, pure hay (Kyle, Male, ESL).

These feelings of embarrassment were confined to RYO tobacco and this inhibited their use:

I think you smoke less when you have rollies because you feel embarrassed rolling in front of other people, but when you have [a pre-manufactured cigarettes brand] you don’t care, you just whip them out (Caitriona, Female, ESL).

A number of negative aspects relating to the products disincentivised RYO use among users including, increasing prices, product issues, the perceived effort associated with rolling the cigarettes, as well as feelings of embarrassment relating to the products occasionally felt by participants.

### Health effects

All of the participants recognised the dangers of smoking, both in terms of smoking effects on themselves and the effects of second-hand smoke on others. Many of the participants perceived themselves to suffer from short-term negative health effects including breathing difficulties and throat problems which they attributed to smoking RYO cigarettes:

I have a very bad throat because of it… but every couple of weeks you would get a bit of a cough and mucus and stuff and its 100% because of rollies (Áine, Female, HES).

Participants accepted that there were long-term adverse health effects of RYO use, acknowledging their awareness and responsibility but expressing less concern because of the temporal remoteness of the potential harmful effects:

I think this is the thing about our generation, there’s no excuse, like every single one of us knows; it’s a future needs problem (Tadhg, Male, HES).

When questioned about their reluctance to quit, they were confident about their ability to do so in the future; however, the immediate benefits within their peer groups disincentivised any short-term intentions of giving up:

I think I’ll give up after college; it’s just part of your lifestyle here (Marcus, Male, HES).

Yet, in many cases, the participants expressed concern over the effects of their smoking practices and second-hand smoke on others. Such concern was particularly clear in relation to vulnerable groups, such as infants, and this often inhibited their smoking behaviours:

I’m not allowed smoke in the house because of my friend’s baby (Davis, Female, ESL).

Many of the participants discussed negative health effects which they believed was attributable to their own RYO use. While concerns relating to the effect of second-hand smoke on others occasionally deterred their smoking practices, the majority of the participants believed that the immediate social benefits outweighed these side effects.

### Denormalisation of smoking

Denormalisation strategies aim to make behaviour less visible in order to reduce its social acceptability [15]. Since 2004, Ireland has introduced various measures focused on denormalising tobacco use such as legislation, health promotion campaigns and policies (Additional file 2). The denormalisation of smoking which has occurred as a consequence of tobacco control policies was identified as a disincentivising factor in RYO use in this study.

An aspect of denormalisation was increased awareness of negative health effects (described above). Another aspect included concern over the perceived reactions of others in terms of young adults’ RYO use. Strong emotions such as fear and guilt were discussed by participants and the presence of such emotions often contributed to a reluctance to use RYO tobacco:

She’s [mother] found multiple packets of rollies and she tries to turn a blind eye, but I don’t smoke in front of her out of respect and it’s a bit of control for me (Áine, Female, HES).

In order to minimise these negative emotions, participants explained how they would often enact strategies of concealment to minimise the chances of ‘getting caught’:

I’ve secret compartments hidden away in my bag. I’m constantly hiding it, never going to show her [mother], never (Sally, Female, HES).

Within workplaces, the participants recognised that the appearance of smoking could be looked upon unfavourably and might be perceived as unhygienic in some professional circumstances, which often led to a curtailment in RYO use:

I work in a kitchen and it’s not an attractive thing to see someone who is working in the kitchen and who is making your food smoking out the front (Áine, Female, HES).

Product negatives, an awareness of the effect of second-hand smoke on others, as well as the fear of judgement from other people regarding their smoking habits comprised the disincentives associated with RYO use.

## Discussion

The declining prevalence of adolescent smoking in Ireland has outstripped the reduction of prevalence in adults. In relation to initiation, for example, some two-thirds of young people have never tried a cigarette compared with only a third twenty years ago [16]. However, RYO use among young people is emerging as a very important impediment to the further reduction of prevalence in this key group, and in attaining government policy of creating a tobacco-free Ireland by 2025. This study found three main factors that incentivised RYO use: intrinsic, social, and environmental factors and confirmed previous findings about smoking and price.

Price is recognised as the most important tobacco control intervention to reduce overall smoking in the population. Young people’s circumstances make them particularly sensitive to price factors, as their relative lack of financial resources incentivises them to seek lowest-cost smoking options. In relation to initiation, this frequently happens through gifting or through accessing parental or familial sources. Once smoking is established, price becomes a dominant consideration. Our findings from adolescent and young adult smokers are consistent with surveys conducted among the general population, with young people in our study reporting price as the most important element determining RYO over pre-manufactured cigarette use [8, 9, 11, 15]. Although the lower price of RYO tobacco is an incentivising factor, we have learned from our qualitative interviews with young people that tobacco control policies resulting in increased taxation and consequent higher pricing is also an effective disincentivising factor. This is a key finding in relation to RYO use in this price-sensitive population, suggesting that government policy, taxation and increased cost have the potential to reduce RYO use and, as a result, overall prevalence in this age group.

However, it’s not all about price, as other product characteristics [taste preferences, user controllability, and beliefs related to the diminished health effects of RYO smoking] [8, 9, 15] are also confirmed. This study extends the work of these previous quantitative studies reporting the reasons behind RYO use, by exploring how young users engage with these products. The ritualistic and artisanal connotations associated with RYO use were found to be a particularly important feature for RYO users, confirming findings from a previous qualitative study [12]. The higher education students in particular discussed how the process of creating the product itself provided satisfaction and was therapeutic, while also giving an opportunity to facilitate social interactions with peers [12]. While none of the early school leavers spoke directly about negative stereotypes [10, 11], some of them discussed feeling “embarrassed” about using RYO among their friends and often managed this embarrassment, not through the instillation of positive attributes in the tobacco products but by concealing their RYO use or by purchasing pre-manufactured cigarettes where possible. Previous studies have reported that RYO use is more common among young people from lower socio-economic groups, [5–7, 10]. While our findings do not negate this, they suggest that this group perceives RYO as an inferior product, used only out of necessity. This suggests a mechanism for influencing RYO use in lower socio-economic groups that would maximise these findings.

Product characteristics were important for incentivising RYO use but cannot be viewed in isolation. Social and environmental factors were powerful influences [16–18] which have not been studied previously in the context of RYO use among young adult users. Peer groups in particular justify the benefits to new users while making brand introductions, providing rolling demonstrations while also strengthening connections between users. Environmental factors such as physical spaces had a role in incentivising RYO use. The assigned and non-assigned ‘smoking areas’, such as within the outdoor areas of licenced premises and the ‘shed’ on a HE campus provided amenities and facilities which provided comfort to users and a space to enact their artisanal routines. These areas also acted as spaces to develop and to sustain friendships.

Disincentivising aspects relating to RYO practices among participants have not been previously explored to our knowledge. Product issues, construction difficulties, and concerns regarding the reactions of others in relation to their smoking habits emerged. Issues relating to the products themselves [mismatch between the rolling materials; soggy filters] were viewed as hampering factors for RYO participants. The participants believed that there were some negative health side effects [breathing difficulties; throat problems] which could be attributed specifically to smoking RYO cigarettes. Yet, the participants perceived the immediate social benefits as outweighing these difficulties.

This study is important as it describes the multidimensional appeal of RYO products. Despite studies being carried out examining the growth of RYO use among adult users, no previous study has provided an in-depth, qualitative account of the incentivising and disincentivising characteristics associated with RYO use among adolescent and young adult smokers.

### Recommendations

Price is the greatest disincentive to smoking in young people as well as in adults. If RYO were equal in price to pre-manufactured cigarettes, then RYO initiation would be much less likely according to our findings. This study has shown that price is just one of a number of incentivising and disincentivising factors in RYO use. Other interventions, such as plain packaging [19–21] and package quantity of tobacco were shown to be important in RYO perpetuation and must be included in any comprehensive plan to reduce RYO use. Many participants in our study believed that RYO is a ‘healthier option’; this false belief should be addressed directly through additional health warnings on RYO products stating that rolling tobacco is as unhealthy as pre-manufactured cigarettes. Educational media campaigns could be used to emphasise this misconception among users. Finally, we know from young people in this study that denormalisation appears to be working, and these efforts should be continued in order to assist in reducing tobacco use among young people.

We recommend the following interventions therefore:

- Increase the price of RYO to match pre-manufactured cigarettes.
- Reduce brand loyalties by introducing plain packaging for RYO. Branding was shown to be particularly important in this study, because of the associations with artisanship.
- Increase RYO pouch size as the unit of purchase. (Eliminating single-cigarette purchase and small cigarette packs is known to have been effective in reducing sales of pre-manufactured cigarettes to young people.)
- Designated smoking shelters in educational and leisure facilities and in other outdoor areas should be abolished. These facilitate smoking in general but are particularly incentivising for RYO use because of the static, shelter and temporal conditions desirable for RYO construction.
- Health warnings specific to RYO use should be used to counteract the misperception that RYO is a “healthier option” than other tobacco products.
- Campaigns to reduce RYO use that capitalise on negative product features could be particularly effective for some groups. For example, the “mess” due to RYO use and people’s embarrassment in using RYO products were emphasised by the early school leavers in our study. Social media campaigns may be especially effective in this regard because of their visual power.
- It is important to address threats of creeping complacency about denormalisation by reinforcing the harms of smoking and the benefits of smokefree legislation.

## Limitations

The first limitation of this study relates to the absence of detailed demographic data about the participants themselves and their smoking habits. Additional information could have been collected (e.g. in questionnaire format) so that differences (e.g. relating to socio-economic status) between the two groups (higher education students and early school leavers) could have been explored fully. As differences based on the socio-economic status of users have been reported in previous studies, this information may have assisted in the framing of our study’s findings.

The second limitation of this study relates to the research approach itself. The study’s qualitative approach was chosen to allow us to examine young people’s RYO use in detail and in depth, unearth their underlying motivations and reasons, and provide a more rounded understanding of RYO use than was heretofore available. We acknowledge the limitations of qualitative approaches in relation to greater dependence on individual researchers’ skills, and added difficulties in maintaining and demonstrating impartiality and rigour, and we took steps to address these as described in the methodology section above, particularly in relation to triangulating authors’ responses to, and interpretation of, the data. Notwithstanding the inherent non-generalisability of qualitative approaches, we hope that the experiences of these two groups may provide transferable illuminating insights about patterns of use among young RYO smokers.

## Conclusions

This study confirms previous findings regarding the importance of price and the prominence of artisanal ritual in explaining RYO use, and adds to these well-established explanations. It provides the first comprehensive qualitative account of RYO use among adolescent and young adults, focussing on both incentivising and disincentivising factors. Specifically, this study identifies social and environmental incentivising factors as well as a range of disincentivising factors (saliently as regards denormalisation) implicated in young people’s RYO use. We have offered recommendations based on our findings and hope that this more nuanced and rounded account will provide scope for the full complexity of RYO use among young smokers to be taken into consideration by those involved in tobacco control initiatives.

## Additional files

Additional file 1: Timeline of Tobacco Control Policies/ Interventions in Ireland, 2000–2016. A table showing the date of introduction and nature of Tobacco Control legislation and other interventions introduced in Ireland from 2000 to 2016. (DOCX 30 kb)

Additional file 2: Interview & Focus Group Guide- May 2016. Instrument used during individual and focus group interviews to facilitate a qualitative exploration of emerging trends in tobacco / nicotine use among young people. (DOCX 20 kb)

## Abbreviations

ESL: Early school leavers
HE: Higher education
HES: Higher education students
MB: The brand smoked by the majority of the participants
PMCs: Pre-manufactured cigarette(s)
Rival brand (1–4): Rival brand (1–4), four other brands smoked by participants
