# Supplementary material for: It’s not all about price: factors associated with roll-your-own tobacco use among young people - a qualitative study
Source: BMC Public Health. 2018 Aug 8;18:991. doi: 10.1186/s12889-018-5921-8 (PMC6083530; doi:10.1186/s12889-018-5921-8)
Supplement: Supplementary file 1 — Timeline of Tobacco Control Policies/ Interventions in Ireland, 2000–2016. A table showing the date of introduction and nature of Tobacco Control legislation and other interventions introduced in Ireland from 2000 to 2016. (DOCX 30 kb) [file 12889_2018_5921_MOESM1_ESM.docx]

**Additional File 1**

**Timeline of Tobacco Control Policies/ Interventions in Ireland, 2000 -2016**
