# Supplementary material for: It’s not all about price: factors associated with roll-your-own tobacco use among young people - a qualitative study
Source: BMC Public Health. 2018 Aug 8;18:991. doi: 10.1186/s12889-018-5921-8 (PMC6083530; doi:10.1186/s12889-018-5921-8)
Supplement: Supplementary file 2 — Interview & Focus Group Guide- May 2016. Instrument used during individual and focus group interviews to facilitate a qualitative exploration of emerging trends in tobacco / nicotine use among young people. (DOCX 20 kb) [file 12889_2018_5921_MOESM2_ESM.docx]

**Additional file 2**

**Interview & Focus Group Guide- May 2016**

***A Qualitative Exploration of Emerging Trends in Tobacco / Nicotine Use among Youth***

# Introductory Questions:

*I’d like to start by just asking you a few questions about yourself, just to get things moving and to get to know you a little bit better.*

Could you start by telling me a bit about your life at the moment?

- - Prompt: Maybe you could tell me a little about your living situation, your work, school? Social life? Family?

What’s a typical weekend like for you?

# Smoking Initiation:

*Now, if it’s ok with you, I’d like to ask general questions about smoking.*

So could you tell me a little bit about the first time you smoked a cigarette?

*PROBES:*

- - Do you remember who you were with?
  - Do you remember where you were?
  - How old were you?
  - Do you remember how you got the cigarette?
  - Do you remember how it felt / tasted?
  - How was that experience?
- Do you remember how / why you decided to smoke a cigarette?
- What was your first impression of smoking?

After that first cigarette, do you remember when you smoked your next cigarette?

*PROBES:*

- - Do you remember who you were with?
  - Do you remember where you were?
  - How old were you?
  - Do you remember how you got the cigarette?
- Was it different from the first time?
- Did you ever/ (or when did you) start smoking regularly?

*PROBES:*

- How do you define regularly?
- How long after smoking their first cigarette did they begin smoking more regularly?
- Approximately how often do you smoke now?

*PROBES:*

When do you smoke the most? During the day? On a night out?

Who do you smoke with?

Is there anywhere you don’t smoke?

Is there anyone you don’t smoke with / in front of?

# Roll-Your-Own Cigarettes (If Relevant):

Can you tell me about the first time you smoked a rollie?

*PROBES:*

Was that the first time you learned about rollies? If not, when was the first time?

Who were you with?

Where did you get the rollies?

How was that experience?

- How often do you smoke rollies now?
- What do you like about rollies?
- What, if anything, don’t you like about rollies?
- Do you like smoking rollies more or less than ‘regular’ cigarettes?
- What are some factors you consider when purchasing rollies?

*PROBES*:

- Why do you like them? Is there a brand preference? How did you come across that brand? Cost? Packaging? Accessibility?

# Marijuana (if relevant)

- Have you ever smoked marijuana?
  - If yes, *PROBE:*
- Can you tell me about the first time you smoked marijuana?
  - - - With whom?
      - Where?
      - How was that experience?
- Do you ever smoke marijuana now?
  - If yes, *PROBE:*
- With who?
- Where?
- How do you smoke? Joints? Pipe? With tobacco?

# E-Cigarettes (If Relevant)

Can you tell me about the first time you used an e-cigarette?

*PROBE:*

- Can you remember where you first heard about e-cigarettes?
- Who were you with the first time you used an e-cigarette?
- Where did you get the e-cigarette?
- How did you find that experience?
- How often do you use e-cigarettes now?
- What do you like about e-cigarettes?
- What, if anything, don’t you like about e-cigarettes?
- What are some factors you consider when purchasing e-cigarettes?

*PROBES:*

- - - Cost? Packaging? Accessibility?
